# Why does the cost of employer-sponsored coverage keep rising?

**DOI:** 10.1093/haschl/qxae078

**Published:** 2024-06-04

**Authors:** Salpy Kanimian, Vivian Ho

**Affiliations:** Department of Economics, Rice University, Houston, TX, 77005, United States; Department of Economics, Rice University, Houston, TX, 77005, United States; Baker Institute for Public Policy, Rice University, Houston, TX, 77005, United States; Baylor College of Medicine, Houston, TX, 77030, United States

**Keywords:** employer-sponsored insurance, payment, provider prices, hospital costs, hospital performance, health insurance, profit margins

## Abstract

Over the past 25 years, the gap between the increase in health insurance costs and workers’ wages has significantly expanded. This trend has led to significant concerns about healthcare affordability, with surveys revealing conflicting opinions regarding whether hospitals or health insurance companies bear the blame for escalating costs. To better understand these dynamics, we examined consumer price indices for health insurance, hospital services, and professional services from 2006 to 2023 using Bureau of Labor Statistics data. Our analysis shows that the hospital price index rose steadily between 2006 and 2023, faster than insurance premiums or professional services. To examine whether differences in underlying costs are driving higher hospital price increases, we evaluated the profit margins of hospitals and health insurance companies using the National Academy for State Health Policy’'s Hospital Cost Tool and National Association of Insurance Commissioners Industry Reports. Our findings reveal that hospitals (for-profit and nonprofit) have consistently maintained higher profit margins than insurance companies. As health insurance costs continue to weigh heavily on working Americans, our analysis suggests that high hospital prices drive insurance premiums.

## Introduction

**Figure 1.**
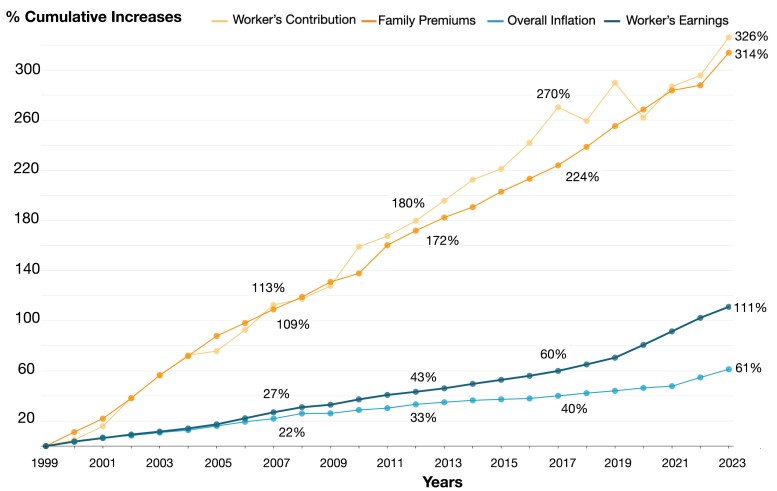
Cumulative increases (%) in workers’ contribution, family premiums, overall inflation, and workers’ earnings, 1999-2023. Cumulative percentage increases were calculated for each component, normalized to 0 in the year 1999. Workers’ contributions and family premiums were obtained from the Kaiser Family Foundation Employer Health Benefits Survey, 1999-2023. Workers’ earnings are from the BLS, seasonally adjusted data from the Current Employment Statistics Survey, 1999-2023. Data are based on the change in total average hourly earnings of production and nonsupervisory employees. Inflation data are from the BLS, consumer price index, historical inflation rates: 1999-2023.

Over the past quarter century, the cumulative increase in the cost of employer-provided family health insurance coverage has grown at a rate more than three times that of workers’ earnings (see Figure 1, which includes data sources and methodology). Meanwhile, cumulative workers’ contributions toward coverage have grown even more (326%) than the overall cost (314%). Moreover, insured workers’ total healthcare spending burden has risen over time as out-of-pocket maximums, deductibles, and other forms of cost sharing have increased.^1^ Not surprisingly, a recent Kaiser Family Foundation survey reported: “About four in ten insured adults worry about affording their monthly health insurance premium, and 48% worry about affording their deductible before health insurance kicks in.”^2^

The growing gap between workers’ earnings and health insurance premiums in Figure 1 leads many to conclude that health insurers are at fault for the healthcare affordability crisis. In the Texas Medical Center’s Consumer and Physician Surveys, 28% of consumers and 47% of physicians blamed rising healthcare costs on insurers, while only 10% of consumers and 9% of physicians blamed hospitals.^3^

Conversely, health economists argue that consolidated hospital systems, or mega providers, are the main driver of rising healthcare spending.^4^ Hospital care reached 5% of gross domestic product in 2022.^5^ Hospitals that enjoy monopoly power as the sole provider in their local market have prices 12.5% higher than markets with four or more hospitals. After hospital mergers occur, prices rise by 6% if the merging hospitals were close neighbors.^6^ A survey administered by the Harvard Opinion Research Program in partnership with the Robert Wood Johnson Foundation and National Public Radio seems more in line with this view. Most respondents agreed that insurance companies (57%) and hospitals (64%) contribute to rising healthcare costs.^7^

## Price indices for hospital and physician care versus insurance

**Figure 2.**
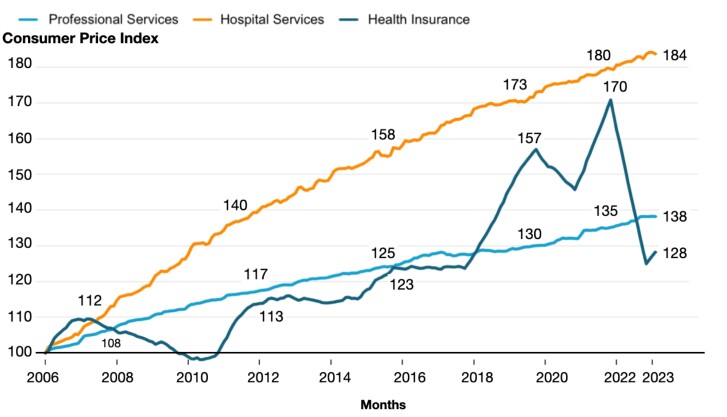
Consumer price index: medical care, by component 2006-2023. Authors’ analysis using data from the BLS, consumer price index, medical care index (by component), 2006-2023.

Rather than comparing the overall rise in insurance premiums to workers’ earnings, Figure 2 shows the comparison of price indices for health insurance, hospital services, and professional services. These data series are sub-components of the all-urban consumer price index compiled by the Bureau of Labor Statistics (BLS). The BLS’ health insurance price index excludes that part of the premium that is used to purchase medical goods and services and instead focuses on the cost to the consumer of purchasing insurers’ services, including risk protection, claim processing, and insurers’ profits.^8^ The retained earnings that the BLS captures using this indirect method therefore do not attribute rising hospital costs to insurers. The hospital services price index includes all services provided and billed by hospitals, including services performed by a physician employed by the hospital. The pricing unit is a hospital visit defined by a specific medical service/diagnosis. The BLS works with respondents to sample each medical service or procedure using a process called sampling by probability proportional to size (PPS).^9^ To calculate the Consumer Price Index (CPI), the BLS selects a sample from the Consumer Expenditure (CE) Survey, which asks respondents where they made purchases.^10^ Hospital services is one of the few items not listed in the CE and sourced from the American Hospital Association Annual Survey by the BLS.^11^ Lastly, the professional services price index represents the services performed and billed by private practice medical doctors, including dentists, eye care providers, and other provider types. The pricing unit in this case is a doctor's visit, which the BLS obtains using CE data by PPS.

For purposes of comparing growth rates over time across services, all prices in Figure 2 were normalized to equal 100 in January 2006, the first year when the BLS began tracking health insurance prices. Figure 2 depicts a significant upward deviation from the trend for the price of health insurance in the year 2018. Between August 2018 and October 2023, the BLS made multiple changes to its methodology for calculating the health insurance price index to widen its representation and improve the timeliness of its calculations, which we list in Figure S1. These changes include a shift toward reliance on National Association of Insurance Commissioners (NAIC) data, addition of Medicare Advantage and Medicare Part D plan data, and use of higher-frequency data. Lastly, since health insurance data are not available in real time and are typically lagged by approximately 10 months, this delay is accounted for in Figure 2, with further details provided in the Supplementary material.

During the pandemic, patients were afraid to visit healthcare providers for non-COVID-related treatment. This decline in healthcare utilization resulted in greater retained earnings, generating substantial profits for health insurers that were widely reported in the media,^12^ which likely contributed to the sharp increase in the health insurance price index that began in 2018. However, once the BLS methodological changes were fully incorporated and patients returned to seeing their healthcare providers, the price index for health insurance also returned to pre-pandemic levels (138 as of July 2022). In contrast, the hospital price index rose steadily between 2006 and 2023, faster than insurance premiums or professional services, reaching its peak (170) in November 2021.

## Inferring the difference between costs and prices using profit margins

It is possible that the hospital price index rose faster, because the cost of inputs used to provide hospital care also rose faster than the inputs necessary for delivering health insurance or professional services. To compare the difference between revenues and costs across sectors, we compared profit margins for insurers and hospitals. We calculated mean hospital net profit margins [(net income)/net patient revenue] by year and ownership type using the National Academy for State Health Policy’s Hospital Cost Tool, which is based on financial information reported to the Center for Medicare and Medicaid Services by hospitals in their annual Medicare Cost Report.^13^ We compared these numbers to health insurance industry profit margins from the NAIC Financial Regulatory Services Department, Insurance Industry Snapshots, and Analysis Reports, which are publicly available.^14^ The NAIC collects this information from all insurers that issue commercial insurance in most states, where the industry is regulated by the state Department of Insurance (except California).^15^

Figure S2 reveals that across all years except for 2022, profits for insurance companies were consistently lower than those for nonprofit and for-profit hospitals. Profit margins were particularly low for insurers between 2014 and 2016 after the insurance provisions of the Affordable Care Act took effect in 2014. The Affordable Care Act (ACA) imposed medical loss ratio requirements on insurers, requiring insurers to issue rebates to customers if their medical spending did not exceed 80% of premiums.^16^ This Medical Loss Ratio (MLR) requirement limited profit margins for insurers, while no such limit was placed on hospitals. The −0.31% net profit margin for nonprofit hospitals in 2022 follows an extraordinarily high net profit margin of 10.67% in 2021. The steep drop is partly attributable to rising labor costs from inflation and the drop in federal funding tied to the end of the COVID-19 public health emergency.

However, a recent analysis found that 85% of financial losses in 2022 for 10 large nonprofit hospital systems were attributable to investment losses,^17^ likely from the fall in the stock market which occurred that year. The Centers for Medicare and Medicaid Services (CMS) requires that hospitals include unrealized investment losses in net income, which is used to calculate net profit margins. For-profit hospitals enjoyed a net profit margin of 7.02% in 2022, which was substantially higher than for nonprofit hospitals and insurers. In the event that a for-profit hospital experiences a shortfall, it can sell more stock to raise cash, an option unavailable to nonprofit hospitals. Therefore, a for-profit system has less need to hold investment assets. While Medicare Cost Reports for all of 2023 are not yet available, hospital financial data from Kaufman Hall for a subset of US hospitals suggest that profits for nonprofit hospitals have rebounded in 2023 with the stock market recovery.^18^

## Conclusion

As the cost of buying health insurance continues to weigh heavily on working Americans, our analysis suggests that high hospital prices are the main driver behind rising premiums. Price increases for hospital care have risen faster than the physician price index or insurance premiums net of medical service costs. Profits for hospitals have also been higher than those for insurers, except for the case of nonprofit hospitals in 2022.

## Supplementary Material

qxae078_Supplementary_Data
