# Supplementary material for: Why does the cost of employer-sponsored coverage keep rising?
Source: Health Aff Sch. 2024 Jun 4;2(6):qxae078. doi: 10.1093/haschl/qxae078 (PMC11195578; doi:10.1093/haschl/qxae078)
Supplement: qxae078_Supplementary_Data [file qxae078_supplementary_data.zip › Supplementary Material-2.pdf]

Documentation

Final numbers are in the accompanying document.

I. Figure 1: Cumulative Increases (%) in Workers' Contribution, Family Premiums, Overall Inflation and Workers' Earnings, 1999-2023

1. Raw Data:

Worker's (Family) Contribution: KFF Employer Health Benefits Survey, 2023
https://www.kff.org/interactive/premiums-and-worker-contributions-among-workers-covered-by-employer-sponsored-coverage/

Family Premiums: KFF Employer Health Benefits Survey, 2023
https://www.kff.org/interactive/premiums-and-worker-contributions-among-workers-covered-by-employer-sponsored-coverage/

Worker's Earnings: Bureau of Labor Statistics and based on the change in total average hourly earnings of production and nonsupervisory employees. Employment, hours, and earnings from the Current Employment Statistics survey, Department of Labor.
https://beta.bls.gov/dataViewer/view/timeseries/CES0500000030

Annual Inflation: Historical Inflation Rates: 2000-2024, Consumer Price Index, Bureau of Labor Statistics
https://www.usinflationcalculator.com/inflation/historical-inflation-rates/

2. Cumulative percentage increases were calculated for each of the above mentioned variables, starting with 0% in 1999 using the following formula:

$$Cumulative\ \%\ increase\ in\ year\ t = \frac{[Variable\ in\ year\ t\ -\ Variable\ in\ year\ 1999]}{Variable\ in\ year\ 1999} * 100$$

II. Figure 2: Consumer Price Index: Medical Care, by Component 2006-2023

1. Raw Data:

BLS's medical care index is composed of medical care services and medical care commodities.

- Medical care services is the larger component in terms of weight in the CPI.
  - It is calculated by tracking consumer out-of-pocket expenses (patient direct payments for medical goods and services and premium).
  - It is divided into professional services, hospital and related services, and health insurance.

- The professional services index covers services that are performed and billed by private-practice medical doctors, dentists, eye care providers, and other medical providers. The pricing unit is a doctor's visit, defined by a specific medical service. At the initial visit, BLS establishes the practitioner's specialty; if it is a group practice, one practitioner is sampled. Then a medical service is sampled via PPS. For Physicians' Services, Current Procedural Terminology (CPT) codes are collected to help describe the item accurately.
- The hospital services index tracks the price of services (both inpatient and outpatient) performed and billed by a hospital or a physician employed by the hospital. The pricing unit is a hospital visit, defined by a specific medical service and diagnosis. At the initial visit, BLS works with the respondent to select a medical service using a process known as sampling by probability proportional to size (PPS). They then document the medical service and specific procedures of the hospital visit.
- The health insurance index covers health insurance premiums. Since it is challenging to control for changes in quality such as policy benefits and risk factors, BLS uses the retained earning approach to calculate this index. Health Insurance index calculation was subject to many changes in terms of data, but in all cases, retained earnings were used, except for a change announced in October 2023 (see Supplementary Figure A1). The retained earnings ratio used in the health insurance index is lagged on average by 10 months. We account for this lag before generating Figure 2.

Note that only the retained earnings ratio for health insurance is lagged. The October 2022 CPI represents 2021 annual retained earnings. For the other components, the October 2022 CPI reflects October 2022 prices.

More detailed technical documentation: https://www.bls.gov/cpi/factsheets/medical-care.htm

To access data, follow https://beta.bls.gov/dataQuery/find?fq=survey:%5Bcu%5D&s=popularity:D and select the variables to download.
U.S. Bureau of Labor Statistics,

Consumer Price Index for All Urban Consumers: Medical Care in U.S. City Average not seasonally adjusted [CUUR0000SAM], retrieved January 26, 2024.

Consumer Price Index for All Urban Consumers: Professional Services in U.S. City Average not seasonally adjusted [CUUR0000SEMC], retrieved January 26, 2024.

Consumer Price Index for All Urban Consumers: Hospital and Related Services in U.S. City Average not seasonally adjusted [CUUR0000SEMD], retrieved January 26, 2024.

Consumer Price Index for All Urban Consumers: Hospital Services in U.S. City Average not seasonally adjusted [CUUR0000SEMD01], retrieved January 26, 2024.

Consumer Price Index for All Urban Consumers: Health Insurance in U.S. City Average not seasonally adjusted [CUUR0000SEME], retrieved January 26, 2024.

2. We normalize January 2006 CPI to 100 for all components and adjust changes accordingly to match changes in the health insurance price index. That is, for all components, $new\ CPI_{January\ 2006} = 100$
   $new\ CPI\_t = new\ CPI_{t-1} + 100 * \frac{old\ CPI_{t-1} - old\ CPI_t}{old\ CPI_t}$ where t is month

III. Supplementary Figure A1: Changes in BLS's Consumer Price Index Methodology for Health Insurance: 2017- present

Source: BLS "Measuring Price Change in the CPI: Medical Care".
https://www.bls.gov/cpi/factsheets/medical-care.htm

IV. Supplementary Figure A2: Net Profit Margins (%): Health Insurance Companies and Hospitals, by Ownership Type, 2011-2022

1. Raw Data:

Hospital Data: The National Academy for State Health Policy's (NASHP) Hospital Cost Tool (HCT)
https://tool.nashp.org/
- Net Profit Margins (%)

Insurance Data: Health Insurance Industry Reports
https://content.naic.org/cipr-topics/insurance-industry-snapshots-and-analysis-reports

2. For hospital data, we take an unweighted average of net profit margin by hospital ownership type and year.
