# Supplementary material for: Why does the cost of employer-sponsored coverage keep rising?
Source: Health Aff Sch. 2024 Jun 4;2(6):qxae078. doi: 10.1093/haschl/qxae078 (PMC11195578; doi:10.1093/haschl/qxae078)
Supplement: qxae078_Supplementary_Data [file qxae078_supplementary_data.zip › Supplementary Figures.pdf]

<u>Appendix Figures</u>

Figure A1: Changes in BLS's Consumer Price Index Methodology for Health Insurance: 2017-present

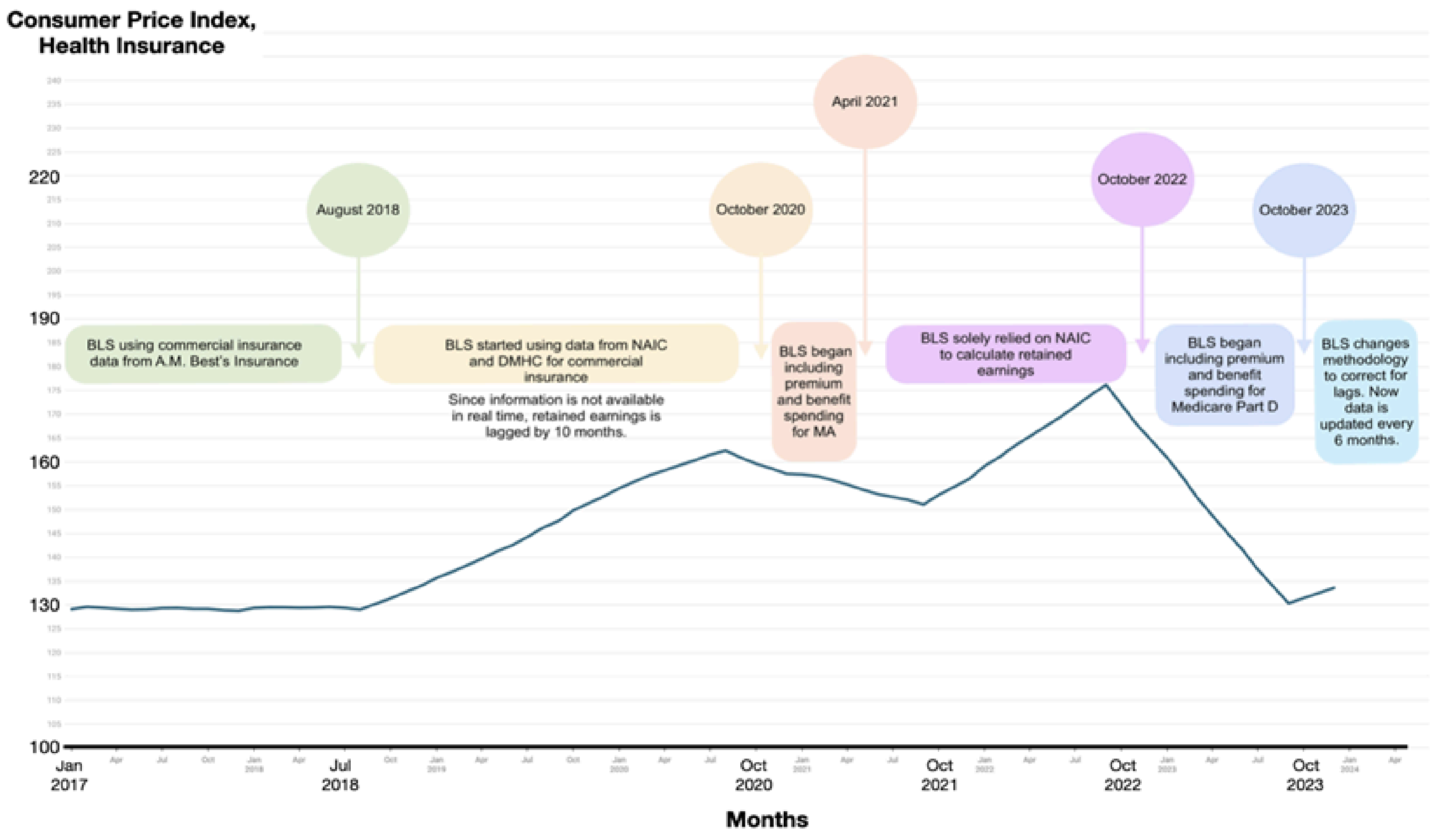

Source: BLS "Measuring Price Change in the CPI: Medical Care".

Figure A2: Net Profit Margins (%): Health Insurance Companies and Hospitals, by Ownership Type, 2011-2022

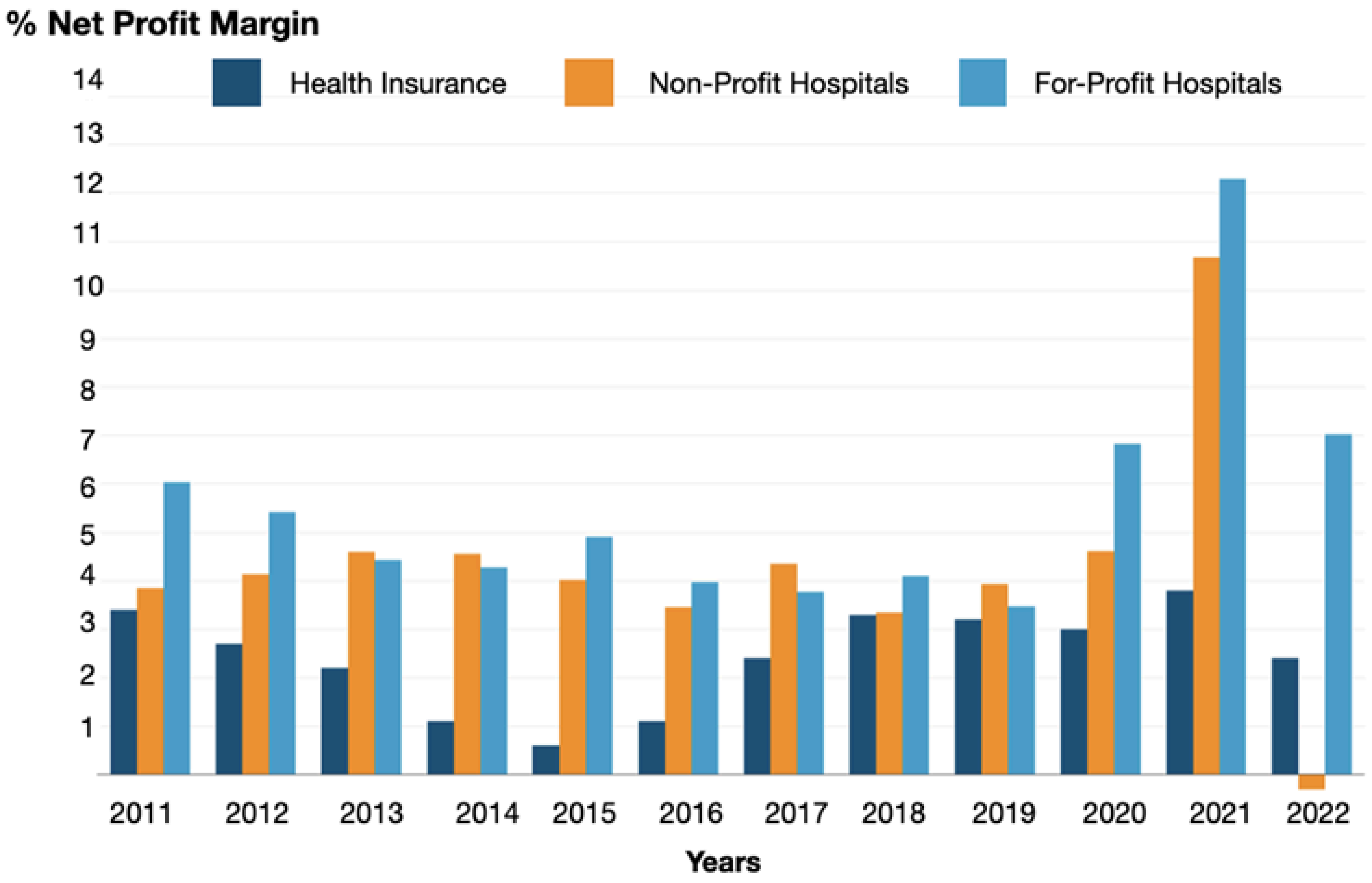

Source: Authors' analysis using data from the National Association of Insurance Commissioners, Financial Regulatory Services Department, Health Insurance Industry Reports, 2011-2022; National Academy for State Health Policy, Hospital Cost Tool, 2011-2022.
